# Prevalence and associated factors of burnout among health sciences students in Spain: a systematic review

**DOI:** 10.47626/2237-6089-2024-0805

**Published:** 2025-04-17

**Authors:** Zoila María Olmos-Bravo, Joan Vicent Sánchez-Ortí, Eugenio H. Grevet, Vicent Balanzá-Martínez

**Affiliations:** 1 University of Valencia Department of Medicine Valencia Spain Department of Medicine, University of Valencia, Valencia, Spain.; 2 Instituto de Salud Carlos III Centro de Investigación Biomédica en Red de Salud Mental Madrid Spain Centro de Investigación Biomédica en Red de Salud Mental (CIBERSAM), Instituto de Salud Carlos III, Madrid, Spain.; 3 INCLIVA Research Institute Valencia Spain INCLIVA Research Institute, Valencia, Spain.; 4 Universidade Federal do Rio Grande do Sul Faculdade de Medicina Departamento de Psiquiatria e Medicina Legal Porto Alegre RS Brazil Departamento de Psiquiatria e Medicina Legal, Faculdade de Medicina, Universidade Federal do Rio Grande do Sul (UFRGS), Porto Alegre, RS, Brazil.; 5 Universidade Federal do Rio Grande do Sul Programa de Pós-Graduação em Psiquiatria e Ciências do Comportamento Porto Alegre RS Brazil Programa de Pós-Graduação em Psiquiatria e Ciências do Comportamento, Universidade Federal do Rio Grande do Sul (UFRGS), Porto Alegre, RS, Brazil.; 6 Hospital de Clínicas de Porto Alegre Programa de Psiquiatria do Desenvolvimento Centro de Pesquisa Clínica Porto Alegre RS Brazil Programa de Psiquiatria do Desenvolvimento, Centro de Pesquisa Clínica, Hospital de Clínicas de Porto Alegre (HCPA), Porto Alegre, RS, Brazil.; 7 HCPA Centro de Pesquisa Clínica Ambulatório de Déficit de Atenção em Adulto Porto Alegre RS Brazil Ambulatório de Déficit de Atenção em Adulto, Centro de Pesquisa Clínica, HCPA, Porto Alegre, RS, Brazil.; 8 University of Valencia Department of Medicine Teaching Unit of Psychiatry and Psychological Medicine Valencia Spain Teaching Unit of Psychiatry and Psychological Medicine, Department of Medicine, University of Valencia, Valencia, Spain.; 9 University of Valencia Valencia Estigma i Salut Mental Valencia Spain Valencia Estigma i Salut Mental (VALSME), University of Valencia, Valencia, Spain.

**Keywords:** Burnout syndrome, prevalence, university students, Spain, risk factors

## Abstract

**Objective:**

There is growing concern about the occurrence of burnout syndrome in university students worldwide. This systematic review aimed to estimate the prevalence of burnout syndrome and its associated factors among health sciences students (HSS) in Spain.

**Methods:**

Five databases (MEDLINE/PubMed, PsycINFO, EMBASE, Dialnet and MEDES) were searched up to January 5, 2023, adhering to PRISMA guidelines. Quantitative studies reporting the prevalence of burnout syndrome among HSS in Spanish universities were considered. The reference lists of the selected studies were hand searched. Data were extracted from peer-reviewed articles.

**Results:**

Twenty-six studies were included with a total of 14,437 HSS. Most studies included nursing students (*k* = 11), followed by medicine students (*k* = 8), psychology students (*k* = 5), dental students (*k* = 2), physiotherapy students (*k* = 1) and pharmacy students (*k* = 1). Overall, study quality was fair. The most widely used instrument was the Maslach Burnout Inventory. The mean prevalence of burnout was 35.3% (*k* = 11 studies). However, rates varied widely between studies, which may be due to methodological differences. Inconsistent associations were found with gender and year of study. The relationship of burnout with academic and mental-health related variables was consistent across studies. Personal attributes, such as higher resilience, are likely protective against burnout.

**Conclusion:**

Burnout appears to be prevalent among HSS in Spain, and may be affected by academic, mental health and personality factors. Identifying risk and protective factors for burnout could help to develop preventive and management strategies to ultimately reduce its negative consequences in this population.

**Systematic review registration:**

PROSPERO (CRD42023387460).

## Introduction

Burnout is a syndrome due to inefficient management of work-related stressors and may involve the development of mental symptoms, physical problems, and increased substance use.^1,2^ Burnout symptoms overlap with common mental symptoms, especially depressive symptoms.^3,4^ The consideration of burnout as a disease is controversial. Indeed, burnout is not a diagnostic category in the DSM-5,^5^ but is classified as a ‘factor influencing health status’ in the ICD-11.^6,7^ Moreover, there is no consensus on the best instrument to measure burnout.^2^

According to some authors, burnout syndrome comprises three main dimensions: emotional exhaustion, depersonalization and lack of personal fulfillment.^8^ Exhaustion is defined as a state of intense fatigue; depersonalization refers to the feeling of detachment or indifference towards clients or patients; and lack of personal fulfillment is defined as the self-perception of ineffectiveness or incompetence at work.^9^ These dimensions are not mutually exclusive, but are often interrelated and can appear sequentially.

Consistent evidence worldwide shows moderate to high levels of burnout among healthcare professionals, including nurses, dentists, physicians, medical trainees, pharmacists, physiotherapists, and psychologists.^10-17^ Burnout can have a negative impact on professionals‘ health and the quality of patient care. For instance, it has been associated with higher risk of self-reported errors among physicians^18^ and worse patient safety.^19,20^

There is growing concern about burnout and mental health problems (MHPs) among university students.^21^ Academic burnout is defined as a feeling of exhaustion due to study demands coupled with a lack of dedication or academic commitment and a feeling of inadequacy as a student.^22^ Academic burnout has been shown to predict subsequent burnout in the work environment.^23^ The development of this syndrome among health sciences students (HSS) may compromise their emotional well-being and academic performance,^24,25^ and can have other negative consequences.^26-28^ Therefore, estimating the prevalence and associated factors of burnout among HSS is relevant. Indeed, burnout is frequent in HSS, such as medical,^29,30^ nursing^31^ and dental students.^32^ However, the way burnout is defined and assessed results in considerable heterogeneity in prevalence estimates.^33^ On the other hand, several risk and protective factors for burnout among HSS have been described, including individual, academic, psychological and social factors.^34-37^ Whether these factors are common or specific across different cultures and university degrees is less researched.

There are several systematic reviews on the prevalence of burnout among students of specific healthcare degrees, namely medicine,^29,30,38^ nursing^31,39^ and dentistry.^32^ However, to our knowledge, no previous review has adopted a comprehensive approach to HSS, including also those enrolled in psychology, pharmacy, and physiotherapy degrees. Moreover, no review has focused on burnout among university students in Spain.

A proper understanding of the prevalence and risk and protective factors of burnout among HSS is needed to develop early intervention, preventive and management strategies in this population, especially for those at risk. These aspects should be studied in each country to tailor prevention and management strategies to a given socio-cultural context. Therefore, this systematic review aims, firstly, to identify the prevalence of burnout in HSS in Spanish universities and, secondly, if sufficient data are available in eligible studies, to identify the factors associated with the development of burnout syndrome. In this review, the terms health science students, healthcare students and health professions students are considered interchangeable.

## Methods

The review was conducted according to the guidelines of the latest version of the Preferred Reporting Items for Systematic Reviews and Meta-Analyzes – PRISMA 2020.^40^ The protocol was registered in the international prospective register of systematic reviews PROSPERO (CRD42023387460).

### Search strategy

The literature search was conducted in five databases: PubMed/Medline, APA PsycINFO, EMBASE, Dialnet and MEDES, with no restriction by date of publication. We used the combination of keywords and MeSH terms "(burnout [OR] "academic burnout" [OR] "emotional exhaustion" [OR] depersonalization [OR] "reduced personal accomplishment") [AND] (university [OR] college) [AND] student [AND] (Spain [OR] Spanish)" to identify records up to January 5, 2023. In MEDES, the following analogous strategy "burnout AND university AND student AND Spain" was used as it allowed a more exhaustive search. In addition, the bibliographic references of the selected studies were reviewed to identify additional studies that met the selection criteria.

### Selection criteria

Studies evaluating the prevalence of burnout in undergraduate students in health sciences degrees (medicine, nursing, dentistry, physical therapy/physiotherapy, psychology and pharmacy) belonging to a Spanish university were included. The results had to provide quantitative data on burnout (prevalence, mean or standard deviation) assessed using a validated scale (e.g., MBI-SS, BCSQ-12-SS; see below). Studies published in English or Spanish were collected. In addition, we only included data reported in peer-reviewed articles, as defined either on the journal website or based on the article full text. In terms of design, we included cross-sectional, cohort, and case-control studies, as well as longitudinal or intervention studies, provided that they reported prevalence data at baseline.

**Figure 1:**
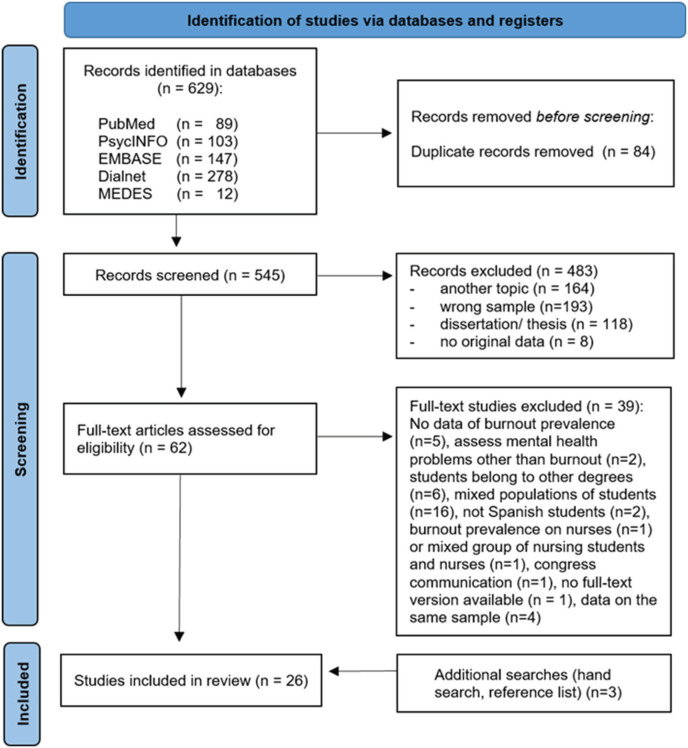
Flowchart showing the identification and selection of studies.

On the other hand, we excluded studies that (1) examined the prevalence of burnout in other populations: students of other university degrees, health professionals or postgraduate students; (2) examined mixed samples of university students without providing disaggregated prevalence data for the group of students in a health science degree; (3) assessed students from countries other than Spain; (4) did not have a design that could be included in a systematic review, e.g., review articles and meta-analyses; case series; opinion articles; dissertations; abstracts of communications to conferences; qualitative research; (5) lacked a full-text version in English or Spanish; (6) assessed MHPs other than burnout; or (7) more than one article provided data on the same sample. The excluded articles and corresponding reasons for exclusion are shown in the flowchart (Figure 1).

Studies were published between 2007 and 2022. Table 1 lists the major characteristics of the reviewed studies: authors and year of publication, year of survey/data collection, sample size, sociodemographic variables (students’ age and female ratio), degree (and year/years of study), response rate, instruments of evaluation of burnout, prevalence of burnout, quantitative values of burnout, MHPs evaluated and factors associated with burnout.

**Table 1:** Major characteristics of the reviewed studies (prevalence and associated factors of burnout among health sciences students in Spain)

References	Year of survey	N	Student's age mean (SD)	Female ratio (%)	Degree (year)	Response rate (%)	Instrument (number of items)	Prevalence of burnout (%)	Mean scores (SD/IQR)	Mental health and personality issues assessed	Factors associated with burnout
Schaufeli^22^	NA	239	22.4 (4)	73	Psychology	NA	MBI-SS (16)		EX 2.48 (1.15) CY 1.72 (1.22) EF 3.76 (0.86)		N/A
Montero-Marin^42^	2011	314	22.05 (3.57)	70.7	Dentistry (1st – 5th)	83.1	MBI-SS (15) BCSQ-12-SS	Huesca: O 28 LD 17 N 19 Santiago: O 19 LD 28 N 24	EX 2.70 (1.50) CY 1.39 (1.18) EF 4.14 (0.94) O 3.32 (1.45) LD 2.46 (1.22) N 2.07 (1.01)		Year of study, academic factors
Montero-Marin^43^	2011	314	22.05 (3.57)	70.7	Dentistry (1st – 5th)	83.1	MBI-SS (15)		EX 13.49 (7.49) CY 5.57 (4.74) EF 24.85 (5.62)	Perceived stress, anxiety, depression, resilience	Perceived stress, anxiety, depression, resilience
Ríos-Risquez^44^	2011	218	24.74 (5.66)	75.7	Nursing (2nd)	100	MBI-GS (16)	EX 28 CY 19.7 EF 25.2	EX 2.43 (1.09) CY 1.65 (1.17) EF 4.23 (0.79)	Resilience	Resilience, working (higher EF), poor relationship with professors (lower EF and EX)
Ríos-Risquez^45^	2014-2016	T1: 218 T2: 113	24.42 (5.27)	75.2	Nursing (T1 2nd, T2 4th)	51.8	MBI-SS (16)		T1 EX 2.43 (1.11) CY 1.67 (1.19) EF 4.32 (0.69) T2 EX 2.40 (1.36) CY 1.45 (1.14) EF 4.21 (0.59)	Psychological distress, resilience	Psychological distress
Moreno-Fernandez^46^	2020	47	20 (2.1, 1.8)	59.58	Pharmacy (2nd)	NA	MBI-SS (11)	63.5 EX 44.6 CY 41.7 EF 60.3	EX 5.26 (1.22) CY 3.11 (1.08) EF 3.25 (1.26)		Intervention (emotional intelligence workshop)
Chust^47^	2014-2015	494	NA	77.7	Nursing (1st, 2nd, 3rd)	68.6	MBI-SS (16)		28.4 (11.2)	Trait anxiety, exam anxiety, sleep satisfaction, self-esteem, life satisfaction	Gender, trait anxiety, exam anxiety, sleep satisfaction, self-esteem, life satisfaction
March-Amengual^48^	2018, 2019	506 (276)*	19.2 (3.06)	64.8	Medicine Nursing Physiotherapy Psychology (1st)	34.2	MBI-SS (15)	6.2 (EX 47.1, CY 7.2)		Psychological distress	Gender, psychological distress
Capdevila-Gaudens^49^	2020	5216	21.4 (3.4)	76.3	Medicine (1 – 6th)	12	MBI-SS (15)	36.8		Depression Anxiety Empathy Substance use	Year of study, depression, trait anxiety, problems of academic performance, lower academic satisfaction, organizational difficulties
Gil–Calderón^50^	2019	1073	NA	75	Medicine (1 – 6th)	NA	MBI-SS (15)		EX 27.5 (7.16) CY 14.83 (7.09) EF 22.38 (6.89)		Gender, year of study, family support, vocation for medicine
Martínez-Rubio^51^	2015-2016	644	22.24 (6.11)	77.3	Nursing Psychology (1st – 4th)	NA	BCSQ-12-SS	Nursing O 25.5 LD 19.7 N 15.6 Psychology O 20.1 LD 25.9 N 18.1		Perceived academic stress	Year of study, mindfulness, self-compassion, psychological flexibility, perceived academic stress factors, living alone
Merino-Godoy^52^	2021	393	23	82.7	Nursing (4th)	NA	ECE (10)		26.28 (7.57)	Resilience, psychological distress	Resilience, psychological distress
Montiel-Company^53^	2013-2014	533	21.9	65.3	Dentistry (3rd, 4th, 5th)	76	MBI-HSS (22)	50.3	56.3		Year of study, degree
		188	22.8	66.7	Medicine (4th, 5th, 6th)			40.4	48.7		
Bresó^54^	NA	193	22.4 (4.2)	73	Psychology	NA	MBI-SS (15)		EX 2.4 (1.1) CY 1.6 (1.1) EF 3.7 (0.8)		N/A
Liebana-Presa^55^	2009, 2010	1009	21.53	85,1	Nursing	NA	MBI-SS (15)		EX 2.6 (1.3) CY 1.2 (1.1) EF 4.1 (0.8)		Gender
Martos^56^	NA	63 (37)*	32.41 (8.48)	69.8	Nursing	NA	MBI-SS (11)		EX 3.83 (1.45) CY 4.01 (0.97) EF 3.94 (0.95)		
Amor^57^	2018	149	21.9 (3.7)	66.4	Medicine (1 – 6th)	87.6	MBI-SS (15)	33.6			Year of study
	2019	224	21.3 (2.4)	69.2		64.4		38			
Atienza-Carbonell^58^	2020	1265	21.4 (3.3)	74.2	Medicine (1 – 6th)	39.3-41.3	IUBA (1)	40.2		Substance use (lifetime and last month)	Gender. Year of study Satisfaction with academic results Number of substances used (lifetime and last month)
Galán^59^	2008	270	NA	71	Medicine (3rd, 6th)	3rd: 65 6th: 35	MBI-SS (15)	22.6 3rd: 14.8 6th: 37.5	Third year: EX 1.8 (0.9) CY 0.6 (0.7) EF 4.4 (0.7) Sixth year: EX 2.4 (0.9) CY 1.4 (1.1) EF 4.1 (0.8)		Year of study
Galán^60^	2009	208	21.8 (3.8)	68.8	Dentistry (2nd, 4th, 5th)	78.8	MBI-SS (15) MBI-HSS (22)	2nd: 41.3 4th: 50.9 5th: 25.6		Depression, suicide ideation	Year of study, depression
Reverté-Villarroya^61^	2017, 2020	305	24	86.5	Nursing (4th)	NA	ECE (10)		30 (23 – 35)	Mental well-being	Completing the degree during the COVID-19 pandemic, mental well-being
Valero-Chillerón^62^	2017	126	22.83 (6.03)	80.2	Nursing (2nd – 4th)	NA	MBI-SS (22)	0			Year of study, satisfaction with clinical practices
Vallejo-Martín^63^	2017	409 (144)*	21.3	68	Nursing (1st – 4th)	NA	SBI-9 (9)		EX 3.13 (1.26) CY 2.10 (1.32) EF 2.93 (1.41)		Gender, degree
Figueiredo-Ferraz^64^	NA	154	21.7 (2.8)	84.3	Psychology (3rd, 4th)	NA	Overload (1) CESQT: exhaustion (4) Disillusion (6)		Overload: 1.9 (0.6) 3rd 2.7 (0.6) 4th Exhaustion: 1.0 (0.6) 3rd 1.7 (0.8) 4th Disillusion: 1.1 (0.6) 3rd 1.5 (0.6) 4th		Year of study, health problems
González-Cabanach^65^	NA	487	21.28 (4.32)	72.7	Physiotherapy (1st, 2nd, 3rd)	NA	MBI (22)		EX 19.6 (10.3) CY 3.5 (4.4) EF 28.9 (8.8)	Self-esteem	Self-esteem
Oro^66^	2013-2015	118	20.25 (1.53)	71.2	Medicine (2nd – 5th)	NA	MBI-SS (15)		5.31 (2.3) EX 2.44 (1.14) CY 0.98 (0.95) EF 4.11 (0.82)	Perceived stress, psychopathology symptoms	Gender, severity of psychopathology symptoms

Studies appear in alphabetical order. Abbreviations. BCSQ-12-SS: Burnout Clinical Subtype Questionnaire Students Survey; CESQT: Questionnaire for the Evaluation of Burnout Syndrome; CY: cynicism; ECE: Emotional Exhaustion Scale; EF: academic efficacy; EX: emotional exhaustion; IQR: interquartile range; IUBA: Single-Item Academic Burnout; LD: lack of development; MBI: Maslach Burnout Inventory; MBI-GS: MBI-General Survey; MBI-HSS: MBI-Human Services Survey; MBI-SS: MBI-Survey for Students; N: neglect; NA: not available; N/A: not applicable; O: overload; SBI-9: School Burnout Inventory; SD: standard deviation.

* In the studies by March-Amengual,^48^ Martos^56^ and Vallejo-Martín^63^ the number of health sciences students is specified in parentheses, since it is a proportion of the joint sample with students of other university degrees.

### Study selection and data extraction

The articles identified in the five databases were imported into the RefWorks platform to determine and eliminate duplicates. Two reviewers (Z.O-B. and J.V.S-O), independently and masked, proceeded to review the titles and abstracts of the articles, evaluating their eligibility according to the selection criteria. In the next step, the reviewers examined the full texts of studies likely to be included in the review to identify eligible studies. In case of discrepancy between the two reviewers, this was resolved by discussion and consensus with a senior author (V.B-M.).

The following data were extracted from each article: authors, year of publication, year of survey, study design, sample size, degree, year of study, gender and age of participants, type of university (public or private), sampling method, response rate, time to data collection, burnout measurement instrument used, prevalence of burnout, scores (means and standard deviations) in the scales and/or subscales of burnout and factors associated with burnout (risk and protective factors).

For studies reporting rates of global burnout and burnout dimensions, mean prevalences were estimated using the following equation: number of individuals with burnout divided by the number of individuals at risk of burnout. For the estimation of the number of individuals with burnout, the percentage of the overall prevalence rate provided in each study was applied to the total number of participants. When studies only reported prevalence rates in each of the burnout dimensions, the same procedure as above was followed, preceded by the calculation of the weighted average of the prevalence rates in each dimension. The latter was used as an estimator of the overall prevalence rate. The number of people at risk of burnout was defined by the total number of participants in each study.

### Study quality assessment

Study quality was evaluated with the National Heart, Lung and Blood Institute (NHLBI) quality assessment tool for observational cohort and cross-sectional studies.^41^ It consists of 14 items, and each item is rated as affirmative, negative, not available or not applicable, and the overall quality of the studies is rated accordingly. Three categories were used to rate study quality: ‘Good methodological quality’, ‘Fair methodological quality’ and ‘Poor methodological quality’.

## Results

### Description of the reviewed studies

A total of 629 records were retrieved from the databases checked: 89 in PubMed/Medline, 103 in PsycINFO, 147 in EMBASE, 278 in Dialnet and 12 in MEDES. The results of the study selection process are displayed on the flowchart (Figure 1). First, duplicate articles (n = 84) were eliminated. After the first screening, based on title and abstract, 483 studies were excluded, because of the reasons shown in the flowchart. Subsequently, the full text of the remaining 62 articles was analyzed, and 39 of them were excluded. Two studies with the same sample^42,43^ and two studies with a partial overlap of the sample^44,45^ were retained because, in both cases, they provided variables of interest that differed from each other. Finally, we included three articles identified in the references of the eligible articles. In summary, of the 629 studies initially located, after eliminating duplicates and applying the selection criteria, 26 eligible articles were included in this systematic review.

In total, there were 14,437 HSS from Spanish universities, enrolled in degrees in medicine (n = 8,581), nursing (n = 3,271), dentistry (n = 1,055), psychology (n = 945), physiotherapy (n = 538) and pharmacy (n = 47). The sample size of the original studies ranged from 37 to 5,216 participants. Most studies (*k* = 23) had a cross-sectional design. Two studies collected data following a longitudinal design, one of which evaluated the evolution of academic burnout over the university years in nursing students^45^ and the other estimated the effects of an intervention on the level of burnout in pharmacy students.^46^

Although the studies were carried out in several regions of Spain, only eleven were multicentric. Studies were conducted at public universities (*k* = 18), private universities (*k* = 2)^47,48^ and both public and private universities (*k* = 4)^49-52^; while one did not specify the type of universities included.^53^

Most participating students were female, representing between 59.6% and 86.5% of the study samples. The average age of participants in the selected studies was between 19.2 and 24.7 years. The years of study are shown in Table 1. Students’ year was not specified in four studies.^21,54-56^

Of the 26 articles, 23 focused on students enrolled in a single degree: nursing (*k* = 9), medicine (*k* = 6), psychology (*k* = 3), dentistry (*k* = 3), physiotherapy (*k* = 1) and pharmacy (*k* = 1). Moreover, three studies recruited students from diverse health sciences degrees: nursing and psychology^51^; medicine and dentistry^53^; and first-year students of medicine, nursing, physiotherapy, and psychology.^48^

Regarding the assessment instruments, burnout was examined in 21 studies using a version of the Maslach Burnout Inventory (MBI),^8^ including the MBI-Students Survey (MBI-SS; *k* = 17),^22^ the MBI-Human Services Survey (MBI-HSS; *k* = 2),^67^ the MBI-General Survey (MBI-GS; *k* = 1),^68^ and the MBI (*k* = 1). The Burnout Clinical Subtype Questionnaire Students Survey (BSQ-12-SS)^69^ and the Emotional Exhaustion Scale (ECE)^70^ were each used twice. The remaining studies employed other validated instruments, such as the Single-Item Academic Burnout (IUBA),^71^ the School Burnout Inventory (SBI)^72^ and the Questionnaire for the Evaluation of Burnout Syndrome (CESQT).^73^ Several studies used more than one instrument.

### Prevalence of burnout

For the 11 studies that reported global burnout rates,^42,44,46,48,49,51,53,57-60^ the mean prevalence was 35.3%. Moreover, five studies^42,44,46,48,59^ reported the mean prevalence of burnout dimensions: emotional exhaustion (41.5%), cynicism (12.9%) and academic effectiveness (31.3%).

Taken together, the prevalence of burnout among medical students ranged from 22.6% to 40.4%.^49,53,57-59^ Regarding dentistry, burnout rates ranged from 25.6% to 50.9% and varied greatly across courses/years.^42,53,60^ The prevalence range was even wider among nursing students.^44,45,47,51,52,55,56,61-63^ For example, Ríos-Risquez et al.^44^ found high levels of emotional exhaustion, high levels of cynicism and low levels of academic effectiveness in 28%, 19.7% and 25.2% of the sample, respectively, whereas another study observed high levels of emotional exhaustion in 17%, but did not identify students with high levels of depersonalization or with low levels of academic effectiveness.^62^ Regarding psychology students, one study provided the prevalence of clinical subtypes of burnout (overload: 20.1%, lack of personal development: 25.9% and neglect: 18.1%),^51^ while three studies reported mean scores.^21,54,64^ In the only study of pharmacy students, 63.5% experienced academic burnout during the COVID-19 pandemic lockdown.^46^ One study showed a medium level of emotional fatigue, low-medium depersonalization, and medium-high personal fulfillment among physiotherapy students.^65^ Finally, March-Amengual et al.^48^ concluded that 6.2% of first-year HSS suffered from burnout.

The two studies^42,51^ that used the clinical subtypes questionnaire (BCSQ-12-SS) in dental, nursing and psychology students, observed a similarly high prevalence of each subtype: overload (19-28%; mean = 20.5%), lack of personal development (17-28%; mean = 19.3%) and neglect (15.6-24%; mean = 15.6%). Moreover, when assessments were confined to one dimension of burnout, moderate levels of emotional exhaustion, assessed with the ECE, were found in nursing students.^52,61^

Instead of reporting burnout prevalence, 14 studies described mean scores on the global burnout scale or its subscales (Table 1). The ranges of these scores vary widely depending on the number of items, the scoring scale and the instrument used, making it impossible to compare scores across studies. Several studies require cut-off points to transform the burnout measure into a dichotomous variable. However, due to the lack of standardized cut-off points, these vary across studies^53,57,66^ or are not reported.^46^ In other cases, percentiles are used as cut-off points, with the first quartile representing the lowest values and the fourth quartile the highest values in each burnout dimension.^44,48,49,59,60,62^ Most of these studies obtained average values -between the second and third quartile- for all three burnout dimensions (Table 1).

### Relationship of burnout with other variables

The reviewed studies examined the association of burnout with sociodemographic variables (gender), year of study, degree, academic-related variables, psychological issues, personality traits, and social support among HSS.

Regarding gender, eight studies found no association with burnout,^42,49,53,56,57,59,60,62^ whereas six studies found that gender was a predictor of burnout or its dimensions. Male students were found to have higher global burnout scores^47^ and higher levels of cynicism,^48,55^ whereas in other studies, female students presented higher levels of global burnout,^58^ emotional exhaustion,^50^ and academic ineffectiveness.^66^

Seven studies found that the prevalence of burnout or its dimensions significantly increased as the year of study progressed.^49,50,51,57,59,62,64^ Conversely, burnout was found to be more prevalent in the preclinical years^58^ or to remain stable throughout the degree.^45^ Among dental students, the highest levels of burnout were observed in the fourth year, with lower levels in the fifth year.^42,53,60^

Three studies analyzed the role of the university degree. Burnout was more prevalent among dental students than medical students.^53^ Moreover, nursing students presented lower levels of cynicism than students of non-health degrees,^63^ whereas burnout levels did not differ between healthcare and non-healthcare students.^48^

Burnout was also associated with several academic-related variables, including academic performance problems, lower academic satisfaction, organizational difficulties, poor relationship with teachers, test anxiety, and objective academic results.^44,47,49,58^ However, burnout levels did not predict academic performance among first year HSS.^48^ Moreover, a higher number of hours dedicated to studying was associated with the frenetic burnout subtype, while a higher number of failed subjects was linked to the negligent subtype.^42,51^ In addition, satisfaction with clinical practice was related to less emotional exhaustion,^62^ and having vocation for medicine when entering university was associated with lower levels of depersonalization and inefficiency.^50^ Finally, nursing students who also worked reported higher levels of personal efficacy, i.e., lower burnout.^44^

Eleven studies analyzed students’ mental health or psychological issues. Burnout was significantly associated with depression,^43,49,60^ anxiety,^43^ substance use,^58^ sleep dissatisfaction,^47^ severity of mental symptoms,^66^ mental well-being^61^ and perceived distress and academic stress.^43,48,51,52^ Moreover, emotional exhaustion was the only burnout dimension that predicted an adverse impact on psychological well-being.^45^

Eight studies examined students’ personality traits and psychological variables. Trait anxiety was associated with burnout.^47,49^ Higher levels of resilience were significantly related to lower emotional exhaustion and cynicism, as well as a greater perception of academic efficacy.^43,44,52^ A longitudinal study observed that students’ level of resilience and psychological well-being increased over time.^45^ Moreover, students’ self-esteem was negatively correlated with academic burnout.^47,65^ In another study, all clinical subtypes of burnout were associated with a lack of psychological flexibility and an absence of self-compassion.^51^

The association between social or family support and burnout was explored in five studies. Family support was identified as a protective factor against burnout in one study,^50^ but not in another.^42^ Moreover, living alone was a risk factor for the underchallenged burnout subtype, while the absence of family support was a risk factor for the negligent subtype.^51^ In two studies, living in the family residence was not significantly associated with burnout.^57,62^

As for intervention studies, an emotional intelligence workshop was shown to have beneficial effects in reducing burnout during confinement due to the COVID-19 pandemic.^46^ In another study examining the relationship between the five facets of mindfulness and the clinical subtypes of burnout, the worn-out subtype was found to have the lowest level of awareness skills.^51^

### Quality assessment

The quality assessment of the studies was rated as fair in 21 studies, good in three and poor in two (Table 2).

**Table 2:** Quality rating of the studies

References	1	2	3	4	5	6	7	8	9	10	11	12	13	14	Total score	Quality rating
Schaufeli^22^	Y	Y	N	Y	N	N	N	NA	Y	NA	Y	NA	NA	N	5/10 (50%)	Fair
Montero-Marin^42^	Y	Y	Y	Y	Y	N	N	NA	Y	NA	Y	NA	NA	Y	8/10 (80%)	Good
Montero-Marin^43^	Y	Y	Y	Y	N	N	N	NA	Y	NA	Y	NA	NA	N	6/10 (60%)	Fair
Ríos-Risquez^44^	Y	Y	Y	Y	N	Y	Y	NA	Y	Y	Y	NA	N	Y	10/12 (83%)	Good
Ríos-Risquez^45^	Y	Y	Y	Y	N	N	N	NA	Y	NA	Y	NA	NA	N	6/10 (60%)	Fair
Moreno-Fernandez^46^	Y	Y	NR	Y	N	Y	Y	NA	Y	Y	Y	NA	Y	N	9/12 (75%)	Good
Chust^47^	Y	Y	Y	Y	N	N	N	NA	Y	NA	Y	NA	NA	Y	7/10 (70%)	Fair
March-Amengual^48^	Y	Y	N	Y	N	N	N	NA	Y	NA	Y	NA	NA	N	5/10 (50%)	Fair
Capdevila-Gaudens^49^	Y	Y	N	Y	N	N	N	NA	Y	NA	Y	NA	NA	Y	6/10 (60%)	Fair
Gil–Calderón^50^	Y	Y	NR	Y	N	N	N	NA	Y	NA	Y	NA	NA	Y	6/10 (60%)	Fair
Martínez-Rubio^51^	Y	Y	NR	Y	N	N	N	NA	Y	NA	Y	NA	NA	Y	6/10 (60%)	Fair
Merino-Godoy^52^	Y	Y	NR	Y	Y	N	N	NA	Y	NA	Y	NA	NA	Y	7/10 (70%)	Fair
Montiel-Company^53^	Y	Y	Y	Y	Y	N	N	NA	Y	NA	Y	NA	NA	N	7/10 (70%)	Fair
Bresó^54^	Y	Y	NR	NR	N	N	N	NA	Y	NA	Y	NA	NA	N	4/10 (40%)	Poor
Liebana-Presa^55^	Y	Y	NR	Y	N	N	N	NA	Y	NA	Y	NA	NA	Y	6/10 (60%)	Fair
Martos^56^	Y	Y	NR	NR	N	N	N	NA	Y	NA	Y	NA	NA	N	4/10 (40%)	Poor
Amor^57^	Y	Y	Y	Y	N	N	N	NA	Y	NA	Y	NA	NA	Y	7/10 (70%)	Fair
Atienza-Carbonell^58^	Y	Y	N	Y	N	N	N	NA	Y	NA	Y	NA	NA	N	5/10 (50%)	Fair
Galán^59^	Y	Y	N	Y	N	N	N	NA	Y	NA	Y	NA	NA	N	5/10 (50%)	Fair
Galán^60^	Y	Y	Y	Y	N	N	N	NA	Y	NA	Y	NA	NA	N	6/10 (60%)	Fair
Reverté-Villarroya^61^	Y	Y	NR	Y	N	N	N	NA	Y	NA	Y	NA	NA	Y	6/10 (60%)	Fair
Valero-Chillerón^62^	Y	Y	N	Y	Y	N	N	NA	Y	NA	Y	NA	NA	N	6/10 (60%)	Fair
Vallejo-Martín^63^	Y	Y	N	Y	N	N	N	NA	Y	NA	Y	NA	NA	Y	6/10 (60%)	Fair
Figueiredo-Ferraz^64^	Y	Y	NR	Y	N	N	N	NA	Y	NA	Y	NA	NA	N	5/10 (50%)	Fair
González-Cabanach^65^	Y	Y	NR	Y	N	N	N	NA	Y	NA	Y	NA	NA	N	5/10 (50%)	Fair
Oro^66^	Y	Y	NR	Y	N	N	N	NA	Y	NA	Y	NA	NA	N	5/10 (50%)	Fair

1. Was the research question or objective in this paper clearly stated?

2. Was the study population clearly specified and defined?

3. Was the participation rate of eligible persons at least 50%?

4. Were all the subjects selected or recruited from the same or similar populations (including the same time period)? Were inclusion and exclusion criteria for being in the study prespecified and applied uniformly to all participants?

5. Was a sample size justification, power description, or variance and effect estimates provided?

6. For the analyses in this paper, were the exposure(s) of interest measured prior to the outcome(s) being measured?

7. Was the timeframe sufficient so that one could reasonably expect to see an association between exposure and outcome if it existed?

8. For exposures that can vary in amount or level, did the study examine different levels of the exposure as related to the outcome (e.g., categories of exposure, or exposure measured as continuous variable)?

9. Were the exposure measures (independent variables) clearly defined, valid, reliable, and implemented consistently across all study participants?

10. Was the exposure(s) assessed more than once over time?

11. Were the outcome measures (dependent variables) clearly defined, valid, reliable, and implemented consistently across all study participants?

12. Were the outcome assessors blinded to the exposure status of participants?

13. Was loss to follow-up after baseline 20% or less?

14. Were key potential confounding variables measured and adjusted statistically for their impact on the relationship between exposure(s) and outcome(s)?

Total score: Number of yes; NA: not applicable; NR: not reported; N: no; Y: yes.

Quality rating: Poor < 50%, Fair 50-75%, Good ≥ 75%.

## Discussion

This systematic review explored the prevalence and associated factors of burnout among HSS from universities in Spain. The 26 studies included 14,437 participants, the vast majority of whom were women, which was expected given the ‘feminization’ of the medical and healthcare workforce.^74,75^ Moreover, the selected studies included students from all years of medicine, nursing, dentistry, and psychology. Overall, the quality of the studies was rated as fair.

### Prevalence of burnout

More than half of participants were medical students, for whom burnout rates ranged between 22.6% and 40.4%. The prevalence of burnout also varied substantially across studies of nursing (17-28%) and dentistry students (25.6-50.9%). Moreover, approximately one out of five HSS presented a clinical subtype of burnout, with overload and lack of personal development being the most prevalent. Estimates of burnout in other health degrees in Spain remain less established. Indeed, none of the studies on psychology students provided standard prevalence rates. Overall, results from HSS in Spain align with recent meta-analyses, which found concerning rates among students in these healthcare degrees. Pooled prevalence estimates of burnout ranged from 37% to 44% for medical students,^29,30^ and about 23% for nursing students.^31^

In the studies that provided burnout rates, these ranged from 0% to 63.5%, with a mean prevalence of 35.3%. This remarkable variability is consistent with that reported in systematic reviews on burnout prevalence among physicians (0% to 85%),^15^ medical students (7% to 75%)^38^ and dental students (7% to 70%).^32^ Such discrepancies may result from the lack of consensus regarding both the definition and assessment of burnout.^3^ Some of the studies reviewed applied the classic three-dimensional definition of burnout, while others chose a two-dimensional definition or measured only emotional exhaustion dimension. This inconsistency is certainly a major weakness in the field. In fact, a meta-analysis found that at least 142 different definitions of burnout were used across 182 studies.^15^ Moreover, ten different assessment instruments were employed in the reviewed studies. Comparing results across studies is challenging, due to differences in questionnaires, number of items, definitions, scoring methods and cut-off scores,^76^ even when considering only versions of MBI questionnaire.^77^

### Factors associated with burnout

The relationship between burnout and students’ gender, year of study and grade (three non-modifiable variables) was inconsistent across studies. The role of age was also found to be inconsistent in meta-analyses of HSS.^29-31^ In most studies, burnout rates increased throughout the years of education, which concurs with previous evidence.^78^ This is particularly concerning when students are transitioning into healthcare professionals, given that burnout has been related to worse healthcare quality and patient safety.^19,20,79^ Moreover, while social and family support were expected to help moderate individual vulnerability to burnout,^36^ the few studies examining these variables also reached inconsistent results.

Notably, the association between burnout and academic-related, mental health-related and personality factors was strong. This is relevant since all three are modifiable risk factors. First, most studies examining academic factors found that burnout was associated with several academic-related variables. Previous evidence suggests that burnout may depend more on factors related to the academic environment and the organization of clinical practices than on individual attributes.^34^ Second, in all eleven studies, several mental health problems were associated with burnout among HSS. Indeed, it is known that burnout syndrome can contribute to the development of mental health symptoms such as anxiety, depression, low self-esteem, insomnia, concentration and memory problems, and increased substance use.^1,2^ There is growing evidence that a substantial proportion of university students suffer from MHPs, particularly depression and anxiety.^80,81^ Similarly, all studies examining students’ personality and psychological factors found significant associations between burnout and self-stem, trait anxiety, and resilience. As expected, a higher level of resilience, conceptualized as the process of adapting effectively in the face of adversity,^82^ acts as a protective factor against burnout.^35^ Overall, the present findings among HSS in Spain concur with meta-analytic evidence supporting the role of educational (e.g., workload, academic satisfaction), and psychological (e.g., self-efficacy and personality traits) factors in burnout among HSS.^31^

### Implications

The substantial rates of burnout among HSS align with the growing concern about the high prevalence of MHPs among university students.^21,83^ The present findings also have several implications for preventing and managing burnout within this population in Spain. This is relevant given the negative consequences of burnout for HSS, including lower levels of professional values and self-concept as healthcare professionals, and dissatisfaction with academic performance.^25-28^

As mentioned above, most associated factors are modifiable. Within the academic environment, strategies such as changes in the grading system, improved accessibility, quality of mental health programs, and mentoring initiatives have been associated with improvements in students’ emotional well-being.^84,85^ Faculties should reflect on possible improvements in their curricula and the organization of clinical practices to promote students’ mental health and emotional well-being.^26^ We recommend that Spanish universities implement policies to change academic conditions in order to reduce the incidence of burnout among HSS.

There is a pressing need to clarify why some students experience burnout while others do not.^86^ Early identification of students at higher risk for burnout should be implemented. This can involve raising awareness of the magnitude of the problem and educating students and faculty to recognize ‘red flags’ (early signs and symptoms) of burnout. Moreover, interventions based on mindfulness, stress management skills, and emotion regulation training could help mitigate the negative effects of burnout on HSS. In this regard, one of the reviewed studies found that increasing students’ emotional intelligence considerably decreased burnout prevalence.^46^ In addition, meditation and mindfulness have been shown to reduce psychological distress and increase empathy in medical students.^87,88^ Strategies aimed at promoting students’ resilience should also take into account the social and structural factors that may influence individual resilience.^82^ Lastly, we support previous recommendations for higher education systems,^39^ such as implementing interventions to promote students’ mental health and sense of competence.

These findings also have some implications for research. Reaching a consensus on the definition of burnout and the assessment instruments is crucial.^15^ Surprisingly, the roles of unhealthy lifestyle behaviours and neuroticism were not assessed in the reviewed studies, despite both being associated with an increased risk of burnout.^31,37^ Further research is needed.

The present systematic review has several limitations. Firstly, not all the reviewed studies aimed to estimate the prevalence of burnout; some were validation studies of burnout assessment tools, e.g., the MBI-SS in dental students.^53^ Secondly, the marked heterogeneity in burnout definitions and assessment methods across studies of the present review precluded the establishment of a pooled prevalence estimate for HSS in Spain. Thirdly, we did not include all health sciences degrees, e.g., podiatry, logopedics. Related to this, extrapolating the results of this review to all HSS in Spain is difficult, due to the unequal representation of the different degrees in the existing studies. Fourthly, most of the included studies had a cross-sectional design, making it impossible to establish causal relationships. More longitudinal and prospective studies are needed to better identify risk and protective factors of burnout in students. Lastly, nine of the articles were published in Spanish. This was expected, given that the topic under review was confined to Spain. Nevertheless, all of them were published in peer-reviewed journals and their quality was not inferior to those published in English.

The strengths of this review include the extensive bibliographic search conducted in five databases. To our knowledge, no systematic reviews have been published addressing burnout prevalence and associated factors among HSS worldwide, making our work a first step in that regard. The present findings provide an overview of these topics in a specific European country. This is relevant, given that burnout prevalence can vary greatly across international literature due to country-specific factors, among other variables.^38^

In sum, our review suggests that burnout is prevalent among health sciences students in Spain, and may be influenced by academic, mental health-related and personality factors. Methodological limitations prevented us from estimating the pooled prevalence of burnout among HSS in Spain. Further research is warranted to identify risk and protective factors for burnout, to ultimately develop preventive and management strategies for this population.
